# Supplementary material for: Effects of Sequential Enzymolysis and Glycosylation on the Structural Properties and Antioxidant Activity of Soybean Protein Isolate
Source: Antioxidants (Basel). 2023 Feb 9;12(2):430. doi: 10.3390/antiox12020430 (PMC9952560; doi:10.3390/antiox12020430)
Supplement: Supplementary file 1 [file antioxidants-12-00430-s001.zip › antioxidants-2091079-supplementary.pdf]

Effects of Sequential Enzymolysis and Glycosylation on the Structural Properties and Antioxidant Activity of Soybean Protein Isolate

Qing Zhang *, Lin Li, Lan Chen, Shuxiang Liu, Qiang Cui, and Wen Qin *

Key Laboratory of Agricultural Product Processing and Nutrition Health of Ministry of Agriculture and Rural Affairs (Co-Construction by Ministry and Province), College of Food Science, Sichuan Agricultural University, No. 46, Xinkang Road, Ya'an 625014, China

*Address correspondence to Qing Zhang, e-mail: zhangqing@sicau.edu.cn; Wen Qin, e-mail: qinwen@sicau.edu.cn

Table S1. Relative MW distribution parameters of F30, F30-10, and F10 [a].

Sample	M_p (Da)				Relative content (%)				M_w/M_n (Peak 2)
	Peak 1	Peak 2	Peak 3	Peak 4	Peak 1	Peak 2	Peak 3	Peak 4	
F30	8044	2640	796	402	5.95	15.05	19.42	59.58	1.18
F30-10	ND	2570	807	392	ND	8.83	18.53	72.64	1.14
F10	ND	1298	825	404	ND	3.55	19.05	77.41	1.11

[a] ND represents no detected. MW, molecular weight; M_p, peak molecular weight; M_w, weight-average molecular weight; M_n, number-average molecular weight.
